# The Student’s Hysteria

**Published:** 1878-02

**Authors:** 


### THE STUDENT'S HYSTERIA.

In a paper on hysteria, which received a prize at the Physical Society of Guy's Hospital this year, Mr. P. Horrocks writes:—

"During the fortnight following the death of the late Napoleon, Sir James Paget was consulted for stone in the bladder, by no less than four gentlemen who had nothing the matter with them. And this leads me to speak of a form of hysteria which is frequent in males, and perhaps more so in our own profession than in any other class of people. How many students are there of one year's standing or more, in this hospital or any other, who have not imagined, and really become convinced, that they were suffering from some disease generally a fatal disease? I, myself, must confess that I have, since coming to Guy's, been thoroughly convinced that my heart was diseased. After a time, however, I felt that I was laboring under a great delusion; it was not my heart, after all, it must be my lungs. I remember listening with breathless attention to Dr. Habershon, as he lectured on phthisis, for I was so convinced that my chest was effected. that I had not, at that time, called up sufficient courage to read it in books, for fear of finding out, without any doubt, that I was a doomed man. One thing, however, I could not get over, and that was that phthisical patients lose their appetites. I have never had that symptom yet, and so, after all, I may only have been suffering from mental delusion. I am not alonein this kind of thing; scores of students consult, yearly medical men, for complaints of which they have not a single symptom. Ask any of our staff; they have had ample experience, and will fully bear out what I have stated."

